# Some Diseases of the Gums: Their Causes and Treatment, with Sketches of Related Incidents in Office Practice

**Published:** 1894-01

**Authors:** D. M. Sabater

**Affiliations:** New York


### SOME DISEASES OF THE GUMS: THEIR CAUSES AND TREATMENT, WITH SKETCHES OF RELATED INCIDENTS IN OFFICE PRACTICE.[1]

BY D. M. SABATER, A.B., D.D.S., M.D., NEW YORK.

THE pathological lesions which are liable to attack the tissues of the gums are very numerous, and, consequently, I will deal only with those of more common occurrence.

They may be conveniently divided into two groups: those with a direct, and those with an indirect origin, or emanating from other than external pathological disturbances, the former due to local, and

---

[1] Read before the Central Dental Association of Northern New Jersey, Meeting, May 15, 1893.

the latter either to local or systemic causes. The direct are, first, gingivitis and ulitis; second, the benign epulis or tumor. The indirect local are: first, cloaca, from sinus in alveolar abscess, the result of devitalized dental pulp caused by various influences; second, perforations with discharge of pus from earthy degeneration, as necrosis, or from fatty degeneration, as caries of bone; these may be local or systemic, the latter form when favoring one of the sequelæ of any of the exanthematous fevers, or from syphilis. "Canker-sores," or cancrum oris, may be local or systemic,—generally the latter. Mucous patches, stomatitis, and pyorrhœa alveolaris are local manifestations of constitutional disturbances.

As is well known, gingivitis marginalis is a temporary condition of hypernutrition of the gingival margin and of the gums in general associated with the ordinary essential phenomena of inflammation,—namely, pain, heat, and swelling, with disturbances of function and nutrition, varying in intensity, extent, and duration. The non-essential phenomenon of inflammation is redness, which, although in this disease it plays an important part, is absent in some cases of inflammation.

*Etiology.*—Gingivitis arises from a direct mechanical or chemical irritation, inducing a disturbance of the cell elements of the parts involved. The principal mechanical and chemical causes are calcareous deposits, the presence of green stains which have strong acid reaction, excessive cleaning, or the use of unsuitable brushes with wide and strong bristles, or deleterious substances found in the composition of some tooth-powders. The use of charcoal and pumice-stone should be condemned not only for their irritating tendency, but also owing to the causing by the former of a well-marked pigmentation or tattooing of the gums.

*Treatment.*—Thorough cleaning by means of scalers of the proper form and size, followed by the judicious use of astringent washes. In severe cases, Church's tr. iodine may be used with antacids, such as bicarb. soda ʒi to aqua ℥viii. In chronic cases, besides the above treatment, I use, first, a four-per-cent. solution of cocaine over the surface of gums, and, five minutes after, a massage of oil of peppermint, oil of camphor, and aristol.

The following is beneficial in gingivitis:

℞ Tr. krameriæ, ℥i;

Tr. opii, ʒii;

Tr. myrrhæ, ʒi;

Aquæ camph., ℥vii.

To be used as a wash.

*Epulis* is a firm tumor of slow growth and variable size, springing from the margin of the periosteum. It often arises between the teeth, and, most commonly, on the labial side. It is either benign or semi-malignant in character; the former is fibrous or granular, and the latter, sarcomatous. The differential diagnosis is made by their manner of growth, their form, and their local manifestations. The fibroid or benign presents a flattened base with a distinct narrow pedicle (mushroom-like), is not extensive, resembles the gums in color, and is of slow growth; while the sarcomatous is sessile, extending into the neighboring tissues. It is of a deep color, presenting a dark-red or purplish spot; bleeds easily when touched, dislodges teeth from their normal position, loosening them by destroying the alveolar sockets.

The *causes* are difficult to determine, especially in the benign form. Its antecedent may be traced to the presence of a local irritation, as badly decayed and broken teeth, roots with ragged edges, or crowded teeth. The semi-malignant epulis, in the majority of cases, is caused by violence and mechanical injuries, as falls, blows, etc.

*Treatment* consists in the removal of the tumor either by ligatures or curved scissors, applying chromic acid or chloride of zinc, sixty grains to one ounce of water, to destroy completely its roots and prevent recurrence; or, better still, operate with the galvano-cautery when applicable. In cases of sarcomatous epulis, the operation consists in the removal not only of the neoplasm, but the surrounding structures, including teeth, alveolus, and periosteum, using chloroform as an anæsthetic.

Actual cautery in cases of persistent hemorrhage and rigid antiseptic measures should be observed, such as solution of bichloride of mercury 1 to 2000, carbolic acid, creoline, hydronaphthol, aristol, iodoform, peroxide of hydrogen, trichloride of iodine, etc. I prefer and use the first mentioned.

*Carcinomatous* or heterologous tumors are sometimes found primarily on the gums. They consist of epithelial cells found in an abnormal situation, and extend deep down into the substance of the gums. The most common is rodent ulcer, or flat cancer, changing to epithelioma. It travels through the system by the lymphatic channels, presenting glandular enlargements. The softer and more juicy the tissues affected, the sooner they are involved; when denser, there is retardation of this process. The younger the patient, the easier and more rapid the infarction of glands. I have seen two cases where there was no enlargement present, one person fifty-five and the other sixty-four years of age. The bed of the excavations presented an unhealthy condition, marked by a distinct, pale, flabby appearance, with everted edges and total absence of granulation. It is generally found in the area of the molar teeth. Its growth is rapid if occurring after middle age,—a characteristic of all malignant tumors,—but if it occurs in early life its growth for many years is slow.

*Causes* are obscure, but they may be traced to a constant and prolonged irritation.

*Treatment.*—The same as in sarcomatous, though more extensive. Should there be a great infarction of the lymphatics and increased symptoms, the operation is of no value, as in these cases the prognosis is generally bad.

*Canker-sore*, or cancrum oris, appears at first as a vesicle, which affects the epithelium to the deeper layers, breaks, and forms a deep ulcer; the base is soft and presents a yellowish or grayish color, and decidedly painful. These lesions attack children more than adults.

*Causes.*—Neglected hygiene of the mouth, unhealthy surroundings, weak and debilitated constitution; in these cases it is well defined, often protracted and extensive; or it may be the result of injuries.

*Treatment.*—Cleanliness, application of nitrate of silver, sulphate of zinc, saturated solution of permanganate of potass., glycero-tannin; and as a wash, chlorate of potass., listerine, or use ℞ Infus. cinchonæ, f℥iss; liquor sodæ chloratæ, f℥ss, to bathe ulcerated surfaces.

*Mucous patches* are syphilitic sores which may be located at any place upon the mucous membrane of the oral cavity. They appear as white ulcers, which become deep and change to a red color, with raised edges and indurated surface and base, being always accompanied by other rational symptoms of syphilis. The difference between these lesions and canker-sores is well established by the evidence of the above symptoms, besides the history of the case.

*Treatment.*—Internally, by the so-called mixed method, consisting of hydrarg. protiodide or bichloride, and potass. iodide. Externally use caustics and antiseptics.

*Stomatitis* is a disease characterized by elevated small patches, like blisters, presenting all the local symptoms of inflammation, and followed by more or less ulceration of the gum's tissue; in some cases it covers a circumscribed territory, and in others often involving the tongue and cheeks.

*Causes.*—Some direct local irritation, as dental caries, extremely warm or cold drinks, corrosive substances, or the consequence of an altered systemic condition, from fevers or impairment of the digestive functions, excessive stimulation or smoking.

*Treatment.*—Immediate removal of sources of irritation, disinfectants and mild caustics on ulcerated surface. Should there be a low tone of health, occasioned by other diseases, give alteratives, tonics, and moderate exercise in the open air. In painful and extensive cases use the following:

℞ Cocaini hydrochloratis, gr. iss;

Sodii chlor., gr. xv;

Glycerinæ, ʒii;

Aquæ, ʒiii.

To be applied on the gums with a camel's-hair brush, and spray frequently with a solution of boracic acid. Internally give bromides.

In cases of fetor of breath, use—

℞ Liq. sodii chlor., ℥iss;

Dec. cinchonæ flav., ℥v;

Mellis rosæ, ℥iss;

Spr. caryophylli, ♏iv.

For mouth-wash.

Pyorrhœa alveolaris, also known as phagedenic pericementitis, Riggs's disease, pyogenic gingivitis, loculis alveolaris, etc., as stated at the beginning of this paper, is a local manifestation of a systemic disturbance, which is due to the action of remedies exhibited for the relief of some constitutional disease. I refer to the preparations of mercury. It is a well-known fact in medical practice that the salivary glands become excessively irritated under the influence of this remedy, and their functional activity is increased to such an extent that an enormous quantity of saliva is secreted and poured into the mouth. It is also well known that ptyalin (organic ferment and active principle of saliva) has a very irritating effect upon the gum tissue, resulting in inflammation which cuts off its nourishment; the result is its detachment from the neck of the teeth, thus forming pockets, which are the chief and constant characteristics of the disease, and are receptacles for particles of food, epithelial scales, fats, etc., forming a mass to be soon infiltrated with lime salts. Here we have the beginning of salivary calculus, which through its irritation of the adjacent periosteum is being constantly added to by the lime salts thrown out by that membrane. This I believe to be the etiology in the majority of cases of true pyorrhœa. In other cases it is due to an early neglect of the teeth, when particles of food are allowed under the margin of the gums. The disease may be circumscribed or diffused, of slow or rapid course, mild or severe in character. In an acute type there is generally a creamy pus discharge, while in the chronic form it is usually watery. In some cases a false abscess is formed at the molar's region, presenting an intense inflammation or discharge and great tenderness of the tooth, so that it may be mistaken for a true alveolar abscess from devitalized pulp. The application of a small piece of ice on the suspected tooth will reveal the true diagnosis. There are other conditions which cause loosening of teeth, as atrophy of roots, a rare occurrence and differentiated from pyorrhœa in the absence of pocket formation and discharge, the gums showing a distinct anæmic condition.

*Treatment* consists principally in thorough scaling and removing of foreign matters, avoiding laceration of gums as much as possible; firm ligatures, to get proper fixation and to avoid movements; also accurate articulation must be obtained; judicious scarification of gums occasionally to relieve congestion, which is always present; removal of offensive decayed roots, and administration of saline cathartics from the onset; strong tonics and most nutritious diet, and the use of antiseptics, alteratives, astringents, and mild caustics. I use, first, hydrogen peroxide with Farrar's syringe, and then warm aqua cinnamomi to clean out pockets. On my second treatment I follow the warm water application with a mild solution of nitrate of silver. The systemic treatment varies, as it must be in accordance with the history of each particular case. In cases of severe pain, use morph. sulph. gr. ii, aqua camph. ℥ii, externally around pocket, and internally chloralamid gr. iii, sodium bromide gr. viii, to be taken in water at bedtime. In cases of great detachment of gums, use mild solution of chloride of zinc.

The latest remedies used in cases of pyorrhœa alveolaris, and which are highly indorsed, are trichloracetic acid and pyrozone.

### PRACTICAL CASES.

*First Case.*—In the spring, 1884, I was called by my friend the late Dr. Clarence H. Young to see one of his patients,—a man about thirty-four years old, apparently in very good health. He complained of smarting sensation around his teeth, especially the front ones. Examination revealed a condition of gingivitis. The doctor, after cleaning several teeth, noticed an unusual bleeding of the gums around each tooth, which he vainly attempted to control by various ways. The hemorrhage was on the inferior jaw, from the central incisors to the first molar, except one bicuspid that was extracted the previous year without hemorrhage. The patient came back next day in the same condition, when he was advised to consult his family physician as to his general condition; he did so, and was found to be suffering with acute Bright's disease of the kidneys, and was placed under treatment at once. The hemorrhage continued for twelve days, gradually decreasing after the fourth day of internal treatment.

*Second Case.*—In November, 1879, Miss D., aged twenty-one, seemingly in good health; her parents living and healthy. On examination of her mouth, I found a small epulis between the left upper lateral and canine teeth. I advised its removal immediately. Bleeding followed, but it ceased in a short time. She had some other work done, leaving the office an hour and a half after in an apparently healthy condition. To my surprise, I was called that evening at ten, as she had a persistent hemorrhage. On arriving at her house, the family physician was doing his best to control the loss of blood. After three-quarters of an hour's work, we decided to use the actual cautery, as the last resort, which answered satisfactorily. The cause of this trouble was the anæmic condition of the patient.

*Third Case.*—On the 15th day of June, 1890, a patient was sent to me by a druggist from Twenty-third Street and Third Avenue. A young man nineteen years old, healthy and strong looking. He complained of excruciating pain in both sides of the face, slight inflammation and rigidity of the masseter muscles, caused by a difficult eruption of wisdom-teeth. I lanced the gums, which was followed by excessive bleeding, which I partially succeeded in controlling, but which did not entirely cease until the next morning, when it was brought under control with tr. ferri persulphas and constant direct pressure by means of cotton, cork, and firm bandages on the face.

*Causes.*—Obscure, probably due to an improper or insufficient fibrin formation.

*Fourth Case.*—In September, last year, Mr. C., thirty-six years old, good constitution and good history of health on previous year. This patient had slight form of pyorrhœa alveolaris. On his first visit I cleaned all the upper teeth, consisting of only twelve, as he had lost two bicuspids and two wisdom-teeth. The operation was attended with a very slight bleeding at the time. Three hours after he returned with his "mouth full of blood," as he expressed it. The oozing was more marked at the molar's region. I had a great deal of trouble in checking said hemorrhage, which I did by carefully packing the pocket around the teeth with fibres of cotton wet with Monsel's solution of iron.

In consultation with J. J. Henna, M.D., of New York, we found a *fibroid tumor* in abdominal wall. The diagnosis was corroborated two days later by Professor Bull of the College of Physicians and Surgeons, and Surgeon to the New York Hospital.

